# Isolation and Quantification of the Hepatoprotective Flavonoids From *Scleromitron diffusum* (Willd.) R. J. Wang With Bio-Enzymatic Method Against NAFLD by UPLC–MS/MS

**DOI:** 10.3389/fphar.2022.890148

**Published:** 2022-06-13

**Authors:** Yuxi Qin, Baojin Zhao, Huifang Deng, Mengjiao Zhang, Yanan Qiao, Qiling Liu, Chuandao Shi, Yunlan Li

**Affiliations:** ^1^ School of Public Health, Shaanxi University of Chinese medicine, Xi’an, China; ^2^ School of Pharmaceutical Science, Shanxi Medical University, Taiyuan, China

**Keywords:** *Scleromitron diffusum* (Willd.) R. J. Wang, flavonoids, the bio-enzymatic method, UPLC-MS/MS, multivariate statistical analysis, hepatoprotective

## Abstract

Flavonoids were the major phytochemicals against hepatic peroxidative injury in *Scleromitron diffusum* (Willd.) R. J. Wang with an inventive bio-enzymatic method by our group (LU500041). Firstly, the total flavonoids from *Scleromitron diffusum* (Willd.) R. J. Wang were extracted by reflux, ultrasonic, ultrasound-assisted enzymatic methods (TFH), and the bio-enzymatic method (Ey-TFH). Then 24 flavonoid compounds were isolated and quantified in the extracts by UPLC-MS/MS. Next, six representative differential compounds in Ey-TFH were further screened out by multivariate statistical analysis compared with those in TFH. In a further step, Ey-TFH presented a higher protective rate (59.30 ± 0.81%) against H_2_O_2_-damaged HL-02 hepatocytes than TFH. And six representative differential compounds at 8 and 16 μmol/L all exerted significant hepatoprotective effects (*p* < 0.05 or *p* < 0.01). Finally, the therapeutic action of Ey-TFH for nonalcoholic fatty liver disease (NAFLD) was processed by a rat’s model induced with a high-fat diet. Ey-TFH (90, 120 mg/kg) significantly ameliorated the lipid accumulation in the rat model (*p* < 0.05). Meanwhile, Ey-TFH relieved liver damage. The levels of ALT, ALP, AST, LDH, and γ-GT in rats’ serum were also significantly reduced (*p <* 0.05 or *p <* 0.01). In addition to this, the body’s antioxidant capacity was improved with elevated SOD and GSH levels (*p <* 0.05) and down-regulated MDA content (*p <* 0.01) after Ey-TFH administration. Histopathological observations of staining confirmed the hepatic-protective effect of Ey-TFH.

## 1 Introduction

Nonalcoholic fatty liver disease (NAFLD) is a metabolic stress liver injury related to insulin resistance (IR) and genetic susceptibility. NAFLD is the most prevalent chronic liver disease worldwide, and its prevalence in adults is estimated at 6.7%–45%. NAFLD is a metabolic syndrome that results in impaired multi-systems (Buzzetti et al., 2016). NAFLD, metabolic syndrome (Mets), and type 2 diabetes mellitus (T2DM) coexist and contribute to cirrhosis, hepatocarcinogenesis, chronic kidney disease (CKD), and colorectal tumors. Clinically, statins have been widely used to treat elevated lipid levels of patients with NAFLD. However, no clear evidence of improving liver fibrosis due to statins has been reported. Obeticholic acid attenuates NAFLD-induced liver fibrosis, but lipid metabolism’s adverse impact limits its application. Beyond these, drugs against liver damage, such as silydianin and bicyclol, have been studied, although the therapeutic effects still need validation in further clinical trials (Friedman et al., 2018; Bessone et al., 2019). Due to the lack of clearly identified treatment targets and regulatory dysfunctions of an organism in NAFLD pathological processes, natural products, which exert therapeutic function by the interaction of multi-targets and multi-ingredients, are potential treatment strategies for NAFLD.

*Scleromitron diffusum* (Willd.) R. J. Wang (syn. *Hedyotis diffusa* Willd., HD, Bai-Hua-She-She-Cao, White-patterned snake’s tongue herb, *Oldenlandia diffusa* (Willd.) Roxd)*,* an herb belonging to the Rubiaceae family, has been clinically used in China for a long time. It has various active phytochemicals such as flavonoids, anthraquinones, terpenoids, sterols, and polysaccharides. The flavonoids as the main bioactive substances of *Scleromitron diffusum* (Willd.) R. J. Wang, exhibit a wide spectrum of pharmacological activities, including hepatoprotective, anti-inflammatory (Zhao et al., 2020), anti-oxidant, anti-viral (Ninfali et al., 2020), anti-diabetic (Mechchate et al., 2020), and anti-peptic ulcer (Serafim et al., 2020) activities. In 2019, the project “Standard of Traditional Chinese Medicinal Materials and Prepared Pieces about *Scleromitron diffusum* (Willd.) R. J. Wang of Shanxi Province” was undertaken by our research team. Upon request, we found the total flavonoids of *Scleromitron diffusum* (Willd.) R. J. Wang extracted by bio-enzymatic method possessed more different active components, lower toxicity, and higher hepatic-protective properties than traditional reflux, ultrasonic, ultrasound-assisted enzymatic methods. In particular, its total flavonoids extracted by intensive bio-enzymatic method received a Luxembourg International Invention patent (LU500041) due to getting more flavonoid types through cellulase. So, this plant was selected to further explore its competitive hepatoprotective activity, material basis, and mechanism of action. In our previous study, Chen et al. have reported the total flavonoids from *Scleromitron diffusum* (Willd.) R. J. Wang protected the liver from oxidative stress injury by inhibiting ASK1/p38 (Li YL. et al., 2020). Tsai et al. have demonstrated that quercetin, a flavonoids compound, could inhibit the M1 macrophage polarization and enhance the M2 macrophage polarization to exhibit anti-inflammatory effects. Moreover, quercetin could be a potential drug to treat neuroinflammation diseases (Tsai et al., 2021). Zhao Y et al. have reported that the total flavonoids from Nakai, a dietary supplement, could significantly remit renal fibrosis in a chronic renal failure animal model (Zhao et al., 2022). With the in-depth studies, many researchers are interested in new flavonoids compounds and their pharmacological activities. In this study, the role of the total flavonoids of *Scleromitron diffusum* (Willd.) R. J. Wang extracted by the bio-enzymatic method (Ey-TFH) in the treatment of NAFLD had been confirmed. Ey-TFH showed a hepatoprotective effect by improving ectopic lipid deposition and antioxidants and alleviating liver impairments.

The total flavonoids of *Scleromitron diffusum* (Willd.) R. J. Wang are gradually becoming a hot spot in research frontiers and extraction methods have attracted more attention. Traditional methods proposed for extracting the total flavonoids include reflux extraction (Zhang R. et al., 2021) and ultrasonic-assisted extraction (Liu et al., 2021; Martín-García et al., 2021; Rezende re al., 2021; Shi et al., 2021). In the last decade, the bio-enzymatic extraction method has been introduced. It is an efficient green technique with several benefits over the conventional methods (Nadar et al., 2018). Enzymes are a kind of bio-catalyst, and proteins are the primary constituents. Because enzymes are usually synthesized in biological cells, they are called bio-enzymes. The bio-enzymatic method is the extraction technique that utilizes bio-enzymes, causing the destruction of plant cell walls and resulting in the outflow of intracellular active ingredients (Zhang X. et al., 2021). Under specific conditions, including the enzyme-substrate concentration, temperature, pH, and reaction time, the higher extraction yields and compounds with more pharmacological activity will be reaped by the bio-enzymatic method. In this work, the total flavonoids of *Scleromitron diffusum* (Willd.) R. J. Wang were extracted by intensive bio-enzymatic method (LU500041). Subsequently, an analytical method combining UPLC-MS/MS and multivariate statistical methods was established to isolate, quantify, and screen the total flavonoids. On the one hand, UPLC-MS/MS, coupling liquid chromatography to mass spectrometry (Li et al., 2011), contributed significantly to the study of TCM over the past few decades (Wiley et al., 2021). Therefore, our research team had used it to simultaneously isolate and quantify flavonoids complex from diverse extracts of TCM, such as *Scleromitron diffusum* (Willd.) R. J. Wang (Chen et al., 2020) and Shuangren-Anshen capsule (Liu et al., 2016). On the other hand, our research group found that the bio-enzymatic method harvests more flavonoids and higher hepatic-protective properties (Li et al., 2016; Li C. et al., 2020) by thoroughly dissolving the cell walls of plants compared with other extraction methods. It is feasible to more competitive total flavonoids using the bio-enzymatic method, and then isolate and quantify them by UPLC-MS/MS. Results showed that compared with the total flavonoids extracted by three common methods (TFH), there are more differential compounds in Ey-TFH. In addition, *in vitro* experiments demonstrated that these differential compounds played vital roles in maintaining the optimal hepatoprotective effect of Ey-TFH.

In summary, the total flavonoids of *Scleromitron diffusum* (Willd.) R. J. Wang had been extracted by the inventive bio-enzymatic method. Next, the bioactivities of Ey-TFH and TFH were compared *in vitro*. Ey-TFH showed a superior effect against NAFLD *in vivo* due to their various pharmacological activities. Our work had further essential implications for flavonoids development.

## 2 Materials and Methods

### 2.1 Herbal Samples and Reagents

*Scleromitron diffusum* (Willd.) R. J. Wang (10 batches) were purchased from Jiangxi and identified by Prof. Tianai Gao from Shanxi Provincial Food and Drug Inspection Institute. Cellulase was obtained from Zhejiang Yinuo Biotechnology Co., Ltd (Zhejiang, China). The 36 flavonoid standards (seen in Table 3) were purchased from Sigma-Aldrich and Steraloids. Fenofibrate, formic acid, methanol, isopropanol, and acetonitrile were HPLC grade from Sigma. Macroporous adsorbent resin AB-8 was obtained from Chengdu Grecia Chemical Technology Co., Ltd. 3-(4,5-dimethyl-2-thiazolyl)-2,5-diphenyl-2-H-tetrazolium bromide (MTT), RMPI 1640 medium, fetal bovine serum (FBS), and trypsin were purchased from Wuhan Servicebio Biological Technology Co., Ltd (Wuhan, China). Triglyceride (TG), total cholesterol (TC), aspartate transaminase (AST), alanine transaminase (ALT)*,* γ-Glutamyltranspeptidase (γ-GT), superoxide dismutase (SOD), glutathione (GSH), lactate dehydrogenase (LDH), alkaline phosphatase (ALP), and malondialdehyde (MDA) assay kits were supplied by Nanjing Jiancheng Bio-engineering Institute (Nanjing, China). Ethanol, methanol, formic acid, and acetonitrile were of chromatographic grade.

### 2.2 Preparation of TFH and Ey-TFH

Dried whole *Scleromitron diffusum* (Willd.) R. J. Wang powders were sieved through a 60 mesh sieve (0.25 mm), degreased by ultrasonic for 30 min, and dried to obtain defatted powders. The conditions of the four extraction methods are shown in Table 1.

**TABLE 1:** Conditions of four extraction methods.

Methods	The bio-enzymatic method	The reflux method	The ultrasound method	The ultrasound -assistant enzymatic
Solution	Ethanol (pH5.0, 50%)	Ethanol (50%)	Ethanol (50%)	Ethanol (pH5.0, 50%)
Cellulase dose	0.5%	-	-	0.5%
Material-liquid ratio	1:30	1:30	1:30	1:30
Extraction time	1.5 h	1.5 h	1.5 h	1.5 h
Temperature	55°C	80°C	-	-

TFH and Ey-TFH were purified by macroporous adsorption resin AB-8 with ethanol (95%, v/v). Next, eluates from AB-8 resin were concentrated at 45°C under reduced pressure and dried. The resulting dry powders were dissolved in a methanol-water solution (7:3, v/v). After vortexing and centrifugation, the supernatants were collected and analyzed by UPLC-MS/MS.

### 2.3 Determination of the Total Flavonoids Yields

The method described by Li YL. et al. (2020) was used to determine the yields of the total flavonoids. First, 0.6 ml of the total flavonoids purified solution was transferred to a 10 ml volumetric flask and mixed with 0.3 ml sodium nitrite (5%, w/v), 0.3 ml aluminum nitrate (10%, w/v), and 4 ml sodium hydroxide (4%, w/v). The final volume of the mixture was adjusted to 10 ml with methanol. Then, the absorbance of the mixture at 500 nm was determined by a UV-vis spectrophotometer (MAPADA instruments Co., Ltd., Shanghai, China). The contents of the total flavonoids were expressed as rutin equivalents through the standard calibration curve (y = 14.07x+0.0116, r = 0.9990). The total flavonoids yields were calculated as follows:

$$\text{Yield }\left(\text{mg}/\text{g}\right)=\frac{\text{the mass of extracted flavonoids }\left(\text{mg}\right)}{\text{the mass of dried sample }\left(\text{g}\right)}$$

### 2.4 UPLC—MS/MS Analysis

#### 2.4.1 Chromatographic Conditions

Waters ACQUITY UPLC I-Class system was used to analyze flavonoids from *Scleromitron diffusum* (Willd.) R. J. Wang quantitatively. The column was HSS T3 (2.5 µm, 2.1 mm × 150 mm) from Waters. The mobile phases consisted of eluent A (0.1% formic acid in water, v/v) and eluent B (0.1% formic acid in acetonitrile, v/v) with a flow rate of 400 μl/min following gradient program shown as follows: 0–3 min, A-B (95:5, v/v); 3–4.3 min, A-B (80:20, v/v); 4.3–9 min, A-B (55:45, v/v); 9–11 min, A-B (2:98, v/v); 11–13 min, A-B (2:98, v/v); 13–15 min, A-B (95:5, v/v). The temperature of the system was set at 4°C. The sample input volume was 5 μl.

### 2.4.2 Mass Spectrometric Conditions

AB SCIEX 5500 QTRAP was used. Selected/multiple reaction monitoring with positive and negative ion switching mode was performed. The ion source parameters of positive ion mode were as follows: Source temperature, 550°C; Gas 1, 55; Gas 2, 50; CRU, 30; ISVF, 5500 V. The ion source parameters of negative mode were as follows: Source temperature, 550°C; Gas 1, 55; Gas 2, 50; CRU, 30; ISVF, -4500V. The data were analyzed using MultQuant and Analyst software.

### 2.4.3 Precision

Equal amounts of all standards were mixed to prepare quality control (QC) samples which were used to ensure the precision of the analytical method.

### 2.4.4 Linear Range

A serial dilution was used to prepare calibration solutions from 125 ng/ml stock solutions of 36 flavonoid standards. Regression lines were calculated by the Least Squares Method. The X-axis showed concentrations (pg/ml) of different solution standards, and Y-axis denoted corresponding peak areas. Correlation coefficient values (R) and linear range were obtained.

### 2.4.5 Determination of Flavonoids Contents

The TFH and Ey-TFH samples were used for UPLC-MS/MS analysis and repeated three times.

### 2.4.6 Multivariate Analysis

The data were imported into SIMCA 14.1 software to analyze differential components of TFH and Ey-TFH. Hierarchical Clustering Analysis (HCA) was used to analyze the difference between each method. Principal component analysis (PCA) was a multivariate statistic method to investigate the correlation of multiple variables. Orthogonal partial least squares discrimination analysis (OPLS-DA) was a supervised multivariate pattern recognition method that was used to screen out potential differential components.

### 2.5 Cell Culture

HL-02 cells (obtained from BOSTER Biological Technology Co., Ltd.) were cultured in RMPI 1640 with 10% FBS, penicillin (100 U/mL), and streptomycin (100 μg/ml) under 5% CO2 at 37°C. When cells were in a logarithmic growth phase, they were treated separately with different concentrations of TFH and Ey-TFH (0, 31.3, 62.5, 125 μg/ml) for 12 h and then exposed to H_2_O_2_ (200 μmol/L). The six flavonoid compounds, including apigenin, chrysin, genistein, isovitexin, naringin, and vitexin (1, 2, 4, 8, 16, 32, 64 μmol/L), were added in the same way as Ey-TFH. The non-treated HL-02 cells were used as the control group.

### 2.6 Protective Rates of Flavonoids

The protective rates for HL-02 cells were determined by the MTT method. The OD was measured, and the protection rates were calculated according to the following equation:

$$\text{Protective rate}\left(\text{\%}\right)=\frac{\text{OD administration}-\text{OD model}}{\text{OD control}-\text{OD model}}\times 100$$

### 2.7 Animal Experiment

For the experiment, 60 rats (provided by the Experimental Animal Center of Shanxi Medical University, No. 22829) were fed adaptively for one week and randomly divided into six groups. Animals were given either a standard chow diet (control group, *n* = 10) or a high-fat diet (HFD, 45% calories from fat, Jiangsu Synergy Medical Bioengineering Co., Ltd., *n* = 50) for three weeks. After successfully modeling, positive control fenofibrate (83 μmol/kg) or Ey-TFH (low-dose, 60 mg/kg; medium-dose, 90 mg/kg; high-dose, 120 mg/kg) solved in distilled water were administrated intragastrically to the assigned HFD groups once every day for two weeks. Meanwhile, the control and model groups were administered the same distilled water (vehicle). Orbital blood and liver tissue of rats were collected and frozen at −80°C for further analysis. According to the manufacturer’s protocols, TG, TC, γ-GT, GSH, SOD, MDA, LDH, AST, ALT, and ALP were investigated in rats’ serum. Rats were killed by decapitation. The livers were quickly separated, washed with saline, and weighed for liver index evaluation. The liver index was calculated according to the following equation:

Liver index=liver weight÷body weight 

Finally, liver tissues were fixed in 4% neutral formalin solution, embedded in paraffin, and were stained with hematoxylin and eosin (H&E). Subsequently, the liver sections were observed under an Olympus iX-51light microscopy (×100).

### 2.8 Statistical Analysis

The data were expressed as means ± standard deviation (SD). Statistical analysis was performed using GraphPad Prism 8.4.0 software (San Diego, CA, United States). A *T*-test was used to compare the differences between groups. *p* < 0.05 was considered significant.

## 3 Result

### 3.1 The Yields of the Total Flavonoids Extracted by Different Methods

The yields of the total flavonoids of *Scleromitron diffusum* (Willd.) R. J. Wang separately extracted by bio-enzymatic, reflux, ultrasonic, and ultrasound-assisted enzymatic methods were shown in Table 2. The yield of total flavonoids from bio-enzymatic was higher than yields obtained by reflux and ultrasonic. The bio-enzymatic method extracted the yield of total flavonoids comparable to the ultrasound-assisted enzymatic method. The results indicated that enzymes played a major role in harvesting more flavonoids.

**TABLE 2:** The yields of the total flavonoids (*n* = 3).

Methods	Yields (mg/g)
Bio-enzymatic method	170.33 ± 2.19
Reflux	154.01 ± 1.19**
Ultrasound method	145.63 ± 1.86**
Ultrasound-assistant enzymatic	168.77 ± 2.99

**p < 0.01, compared with the bio-enzymatic method.

### 3.2 Establishment of UPLC-MS/MS Method

**FIGURE 1:**
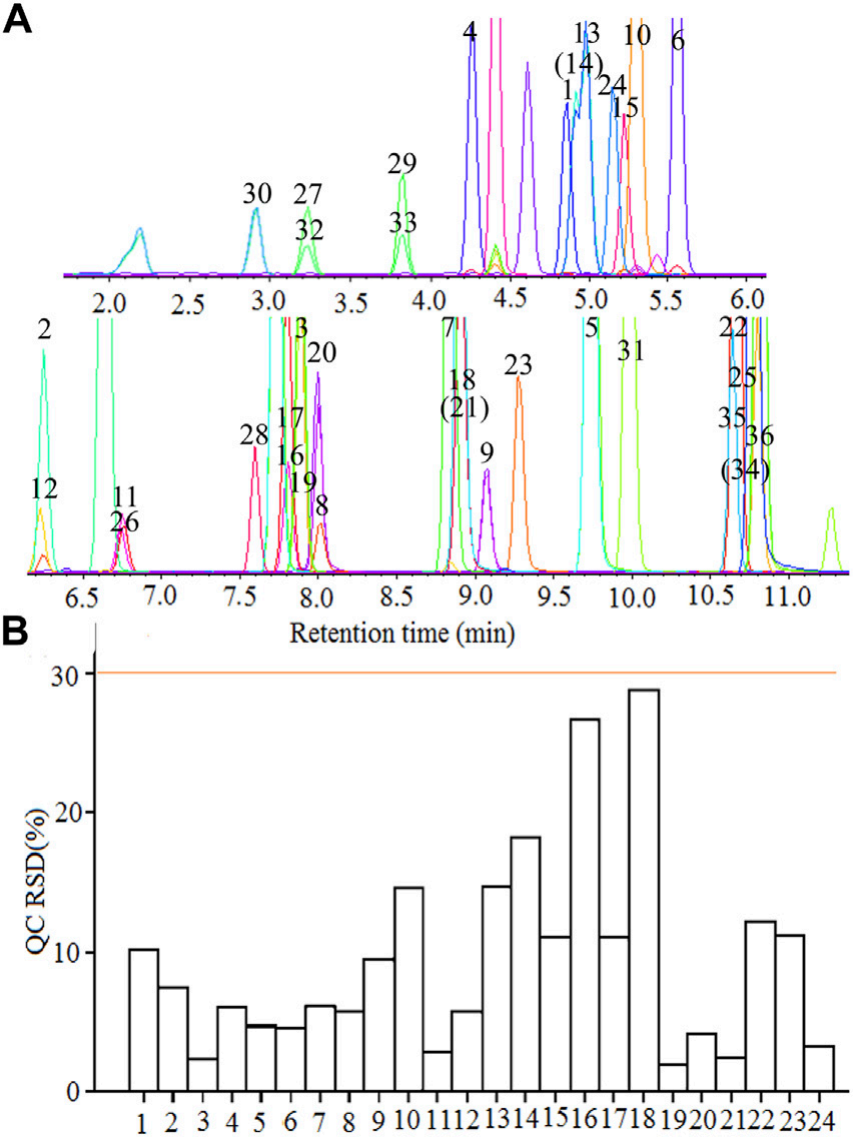
UPLC-MS/MS determination of flavonoid compounds. **(A)** The extracted ion chromatograms (XIC) of 36 flavonoid standards; **(B)** The relative standard deviation (RSD) of 24 representative quality control (QC). 1 Rutin, 2 Naringin, 3 Glycetein, 4 Daidzin, 5 Isoliquiritigenin, 6 Genistin, 7 Naringenin, 8 Luteolin, 9 kaempferol, 10 Taxifolin, 11 Myricetin, 12 Quercitrin, 13 Vitexin, 14 Isovitexin, 15 Isoquercetin, 16 Liquiritigenin, 17 Daidzein, 18 Genistein, 19 Eriodictyol, 20 Quercetin, 21 Apigenin, 22 Chrysin, 23 Isorhamnetin, 24 Luteolin-7-O-glucoside, 25 Biochanin A, 26 Butin, 27 Catechin, 28 Dihydrokaempferol, 29 Epicatechin, 30 (-)-Epigallocatechin, 31 Formononetin, 32 (+)-Gallocatechin, 33 Glycitin, 34 Sakuranetin, 35 Kaempferide, 36 Puerarin.

The contemporaneous quantification of 36 flavonoid compounds was validated. The extracted ion chromatograms (XIC) of 24 representative flavonoid standards are shown in Figure 1A. The retention time, mass information, standard curves, and concentration range of 36 flavonoids standards are shown in Table 3. As seen in Figure 1A and Table 3, each component could be well separated, and the peak shape was sharp and symmetrical. High correlation coefficient values (R^2^ > 0.99) were obtained, indicating strong linearity at a relatively wide range of concentrations. The relative standard deviation (RSD) of 24 representative QC samples is shown in Figure 1B. The precisions were < 30%. The obtained results demonstrated that this UPLC-MS/MS method met the methodological requirements.

**TABLE 3:** The retention times, mass information, standard curves, and concentration range of 36 flavonoid standards.

Compound name	R_t_ (min)	Mass information	Linear	Correlation coefficient R^2^	Concentration range (ng/ml)
Apigenin	8.90	269.0/117.0	Y = 12.630X + 74927.3	0.9984	0.0125-125
Biochanin A	10.65	283.0/268.0	Y = 7.082X + 83735.7	0.9967	0.0125-62.5
Butin	6.97	271.0/135.0	Y = 18.160X + 93542.3	0.9997	0.625-125
Catechin	3.26	289.0/245.0	Y = 1.591X + 9661.2	0.9974	0.125-125
Chrysin	10.64	253.0/143.0	Y = 4.810X + 1997.9	0.9996	0.0625-125
Daidzin	4.26	417.0/255.0	Y = 7.03–X−16753.5	0.9990	0.025-125
Daidzein	7.64	255.0/199.0	Y = 2.35–X−16935.1	0.9995	0.125-125
Dihydrokaempferol	6.76	289.0/153.0	Y = 1.16–X−11745.4	0.9967	0.25-125
Epicatechin	3.73	289.0/203.0	Y = 0.862X + 660.2	0.9968	0.125-125
(-)-Epigallocatechin	2.76	305.0/125.0	Y = 1.595X + 15392.1	0.9976	0.25-125
Eriodictyol	7.81	289.0/153.0	Y = 5.98–X−11739.2	0.9994	0.025-125
Formononetin	9.99	267.0/223.0	Y = 14.070X + 63221.9	0.9996	0.025-125
(+)-Gallocatechin	3.33	305.0/125.0	Y = 1.690X + 6243.1	0.9963	0.0625-125
Genistin	5.50	433.0/271.0	Y = 8.898X + 50000.2	0.9993	0.0625-62.5
Genistein	8.91	271.0/153.0	Y = 7.830X + 100786.0	0.99646	0.025-125
Glycetein	7.88	283.0/268.0	Y = 6.306X + 11582.2	0.9988	0.0625-125
Glycitin	4.05	447.0/285.0	Y = 10.976X + 40486.2	0.9986	0.0125-62.5
Isoliquiritigenin	9.74	255.0/119.0	Y = 26.255X + 10532.6	0.9999	0.125-125
Isoquercetin	5.23	465.0/303.0	Y = 3.303X + 46287.9	0.9969	0.25-125
Isorhamnetin	9.28	317.0/302.0	Y = 4.42–X−12105.9	0.9986	0.25-125
Isovitexin	4.99	431.0/311.0	Y = 8.82–X−10218.1	0.9996	0.0125-125
Kaempferide	10.42	299.0/284.0	Y = 19.297X + 478094	0.9968	0.125-125
Kaempferol	9.07	287.0/153.0	Y = 2.16–X−3130.7	0.9999	0.625-125
Liquiritigenin	7.74	255.0/135.0	Y = 13.305X + 92629.4	0.9981	0.125-125
Luteolin	8.00	287.0/153.0	Y = 4.19–X−15833.1	0.9999	0.25-125
Luteolin-7-O-glucoside	5.31	449.0/287.0	Y = 10.73–X−21914.4	0.9997	0.125-125
Myricetin	6.88	319.0/153.0	Y = 0.96–X−3536.7	0.9962	2.5-125
Naringin	6.23	579.0/271.0	Y = 1.31–X−2461.8	0.9990	0.025-125
Naringenin	8.84	271.0/151.0	Y = 13.731X + 70911.8	0.9986	0.0625-125
Puerarin	10.81	285.0/242.0	Y = 5.06–X−46492.2	0.9995	0.0125-125
Quercetin	8.02	303.0/153.0	Y = 1.127X + 12670.6	0.9990	1.25-125
Quercitrin	6.25	447.0/301.0	Y = 4.42–X−17750.7	0.9992	0.0125-125
Rutin	4.86	611.0/303.0	Y = 3.869X + 6174.8	0.9965	0.025-125
Sakuranetin	10.25	287.0/167.0	Y = 14.245X + 70551.3	0.9970	0.00125-125
Taxifolin	5.44	305.0/153.0	Y = 0.98–X−18415.9	0.9983	1.25-125
Vitexin	4.99	431.0/311.0	Y = 8.40–X−26598.5	0.9997	0.025-125

### 3.3 Determination of the Content of Flavonoids

The quantitative determination of 24 representative flavonoids components in TFH and Ey-TFH are shown in Table 4 (Supplementary Sheet S1). The total levels of flavonoids in the studied extracts ranged from 201 to 207 ng/g. There was no difference between TFH and Ey-TFH in contents. In sharp contrast, compared with TFH, the contents of six flavonoids components (genistein, isovitexin, luteolin, luteolin-7-O-glucoside, naringin, and vitexin) were higher in Ey-TFH (*p < 0.05* or *p < 0.01*). Furthermore, Ey-TFH contained these three components (apigenin, chrysin, and dihydrokaempferol), which were not detected in TFH.

**TABLE 4:** Determination of flavonoids components’ contents (*n* = 3).

Flavonoid components	Contents (ng/g)			
	Bio-enzymatic extraction	Reflux extraction	Ultrasound extraction	The ultrasound -assistant enzymatic
Apigenin	555.4 ± 22.7	-	-	-
Chrysin	201.3 ± 25.8	-	-	-
Daidzin	67.7 ± 6.5	152.2 ± 20.3	123.9 ± 2.4	116.9 ± 13.0
Daidzein	360.9 ± 20.7	348.4 ± 37.7	348.5 ± 29.2	376.2 ± 34.6
Dihydrokaempferol	150.0 ± 31.4	-	-	-
Eriodictyol	107.8 ± 5.9	371.3 ± 5.1	357.7 ± 16.9	349.3 ± 9.9
Genistin	75.1 ± 20.0	104.8 ± 26.0	96.4 ± 9.3	92.2 ± 9.3
Genistein	1359.0 ± 373.6	119.8 ± 42.3**	122.1 ± 7.6**	140.2 ± 19.0**
Glycetein	2462.0 ± 525.1	2066.0 ± 221.6	1745.0 ± 64.9	1891.0 ± 77.8
Isoliquiritigenin	-	4.4 ± 1.2	4.5 ± 0.4	6.2 ± 0.9
Isoquercetin	64649.0 ± 10716.0	63326.0 ± 4412.0	72141.0 ± 1840.0	71356.0 ± 1514.0
Isorhamnetin	456.4 ± 167.8	515.8 ± 118.5	439.9 ± 47.4	489.0 ± 77.5
Isovitexin	806.0 ± 54.5	116.9 ± 4.6**	122.9 ± 7.1**	109.9 ± 6.2**
Kaempferol	3300.0 ± 611.2	4496.0 ± 1263.0	4070.0 ± 513.3	3845.0 ± 753.1
Liquiritigenin	-	14.2 ± 1.5	15.3 ± 4.7	39.5 ± 0.8
Luteolin	1092.0 ± 355.4	363.2 ± 71.1*	391.2 ± 4.6*	394.3 ± 50.8*
Luteolin-7-O-glucoside	986.5 ± 139.9	389.3 ± 38.9**	405.3 ± 24.7**	379.7 ± 3.7**
Myricetin	228.8 ± 35.3	237.3 ± 50.0	254.3 ± 17.6	260.9 ± 76.4
Naringin	280.8 ± 40.7	77.2 ± 1.5**	63.4 ± 8.8**	125.8 ± 6.9**
Naringenin	49.7 ± 7.9	96.3 ± 7.0	88.7 ± 3.9	98.7 ± 5.5
Quercetin	17987.0 ± 2348.0	18284.0 ± 3117.0	13828.0 ± 1176.0	14206.0 ± 2175.0
Quercitrin	4935.0 ± 493.3	2868.0 ± 154.0	2787.0 ± 246.7	2877.0 ± 291.2
Rutin	102284.0 ± 4680.0	105461.0 ± 8028.0	103026.0 ± 1723.0	108999.0 ± 1062.0
Taxifolin	1571.0 ± 208.6	914.3 ± 192.5	945.8 ± 127.2	957.4 ± 53.9
Vitexin	825.5 ± 51.2	135.5 ± 3.8**	131.2 ± 9.2**	126.0 ± 4.7**
The total flavonoids	204722.1 ± 5929.0	200485.4 ± 14416.0	201506.9 ± 5327.0	207236.7 ± 3148.0

*p < 0.05, **p < 0.01, compared with the bio-enzymatic method.—represents non-detectable.

### 3.4 Differential Components Analysis

#### 3.4.1 HCA Analysis

**FIGURE 2:**
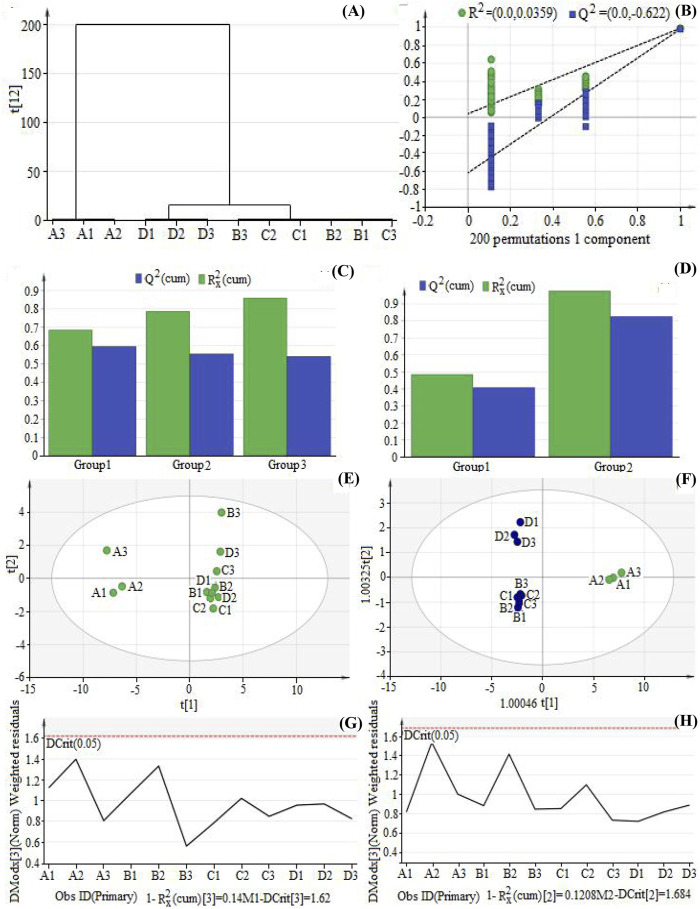
Hierarchical clustering analysis (HCA) analysis, Principal component analysis (PCA) analysis, and Orthogonal partial least squares discrimination analysis (OPLS-DA) analysis. **(A)** The diagram of HCA; **(B)** The result of permutation text of OPLS-DA; **(C)** The fitting result of PCA; **(D)** The fitting result of OPLS-DA; **(E)** Score plot of PCA; **(F)** Score plot of OPLS-DA; **(G)** Distance to model (DModx) plot of PCA; **(H)** DModX Plot of OPLS-DA. A1, A2, A3 represent bio-enzymatic method; B1, B2, B3 represent reflux method; C1, C2, C3 represent ultrasonic method; D1, D2, D3 represent ultrasound-assisted enzymatic method.

Multivariate statistical analysis was performed to screen the differential components among TFH and Ey-TFH. HCA was a process of dividing data into different classes or clusters. When the analysis was reasonable and accurate, similar samples of the same group could appear in the same cluster through clustering, while samples of different clusters had specific differences. As shown in Figure 2A, assuming that a suitable level of distance was selected, extracts prepared by four methods could be divided into two main clusters: bio-enzymatic extracts were cluster 1; the other three extracts (reflux, ultrasonic, and ultrasound-assisted enzymatic methods) were cluster 2. Namely, Ey-TFH were cluster 1 and TFH were cluster 2. The differences could be intuitively seen from the distance of samples in the cluster analysis diagram. There was a significant difference between the bio-enzymatic method and the other three extraction methods.

### 3.4.2 PCA Analysis

In order to investigate the differences among the four preparation methods, a widely used tool, PCA, was applied. The results were shown in Figures 2C,E,G. According to PCA relevant results (Figure 2E), three principal components were included as the optimal number of latent variables. *R* ^2^_X_ and *Q* ^2^ were important parameters of the PCA model. The quality of the model was described by the *Q* ^2^ and *R* ^2^ (*R* ^2^_X_ or *R* ^2^_y_) parameters. *Q* ^2^ denoted the predictability, and *R* ^2^ indicated the goodness of fit. In this study, *R* ^2^_X_ = 0.86, *Q* ^2^ = 0.543. Both parameters were greater than 0.5, and all the samples fell within the 95% confidence interval, suggesting that the model was reliable. The samples could be classified into two separate categories based on the PCA scores plot (Figure 2E). Samples of bio-enzymatic method (A1, A2, A3) belonged to category 1, while samples of reflux method (B1, B2, B3), ultrasonic method (C1, C2, C3), and ultrasound-assisted enzymatic method (D1, D2, D3) belonged to category 2. That is to say, Ey-TFH belonged to category 1, while TFH belonged to category 2. That implied dissimilarity between Ey-TFH and TFH. According to Figure 2C, the overlaps among the samples of reflux method (B1, B2, B3), ultrasonic method (C1, C2, C3), and ultrasound-assisted enzymatic method (D1, D2, D3) were apparent. In other words, there was no significant difference between these three methods. Distance to model (DModX) was applied to measure mismatch with normal. As shown in Figure 2G, the DmodX values of all samples were within the control limits, indicating that all samples had reliable quality.

### 3.4.3 OPLS-DA Analysis

OPLS-DA was also conducted to explore differential components contributing to the group separation. OPLS-DA was a kind of supervised pattern recognition method. It was designed to remove the variation not associated with the response being studied. An improved level of group classification and a better understanding of the variations responsible for classification was constructed. A permutation test was adopted to reveal overfitting (Figure 2B). All the left points of R^2^ and Q^2^ were lower than the rightmost original ones. The vertical axis intercept by the regression curve of Q2 points was less than zero. These indicated that this OPLS-DA model was reliable and free from overfitting. There were two principal components, as shown in the OPLS-DA fitting results (Figure 2D). The *R* ^*2*^ and *Q* ^*2*^ values were nearly close to 1 (*R* ^2^_X_ = 0.88, *R* ^2^_Y_ = 0.98, *Q* ^2^ = 0.87), suggesting the model was validated and considered highly relevant. Greater *R* ^*2*^ and *Q* ^*2*^ values indicated that OPLS-DA analysis could better reflect between-group differences. Figure 2F shows that the samples of the bio-enzymatic method (A1, A2, A3) were distributed into a cluster distinct from those of the reflux method (B1, B2, B3), ultrasonic method (C1, C2, C3), and ultrasound-assisted enzymatic method (D1, D2, D3). Namely, Ey-TFH was distributed into a cluster distinct from TFH. Similar to the PCA loading plot, the separated distributions implied discrimination between TFH and Ey-TFH. Also, OPLS-DA was able to separate the samples of reflux, ultrasonic, and ultrasound-assisted enzymatic methods, which were overlapped completely in the PCA loading plot. The DmodX value of all samples (Figure 2H) showed that there were no abnormal points, demonstrating that the data were reliable.

### 3.5 Screening for Differential Compounds of TFH and Ey-TFH

Table 5 illustrates the variable importance in projection (VIP) values of the OPLS-DA model. The greater VIP value, the stronger contribution to the model explaining. VIP score >1 was usually used as a threshold. VIP value confirmed the assessment results of differential components, as shown in section 3.3. For three compounds only found in Ey-TFH, contents shown in Table 4 and VIP values of apigenin and chrysin were higher than those of ihydrokaempferol. Taking both aspects into consideration, apigenin and chrysin, but not ihydrokaempferol, were selected for the subsequent *in vitro* experiments.

**TABLE 5:** The components with variable importance in projection (VIP) values greater than one as threshold.

Number	Flavonoids components	VIP
1	Chrysin	1.20162
2	Vitexin	1.19987
3	Apigenin	1.19658
4	Isovitexin	1.19621
5	Eriodictyol	1.19584
6	Dihydrokaempferol	1.19395
7	Luteolin-7-O-glucoside	1.18618
8	Genistein	1.18319
9	Quercetin	1.17531
10	Naringenin	1.14327
11	Naringin	1.14004
12	Isoliquiritin	1.10812
13	Luteolin	1.08441
14	Dihydroquercetin	1.07211
15	Daidzin	1.03702

**FIGURE 3:**
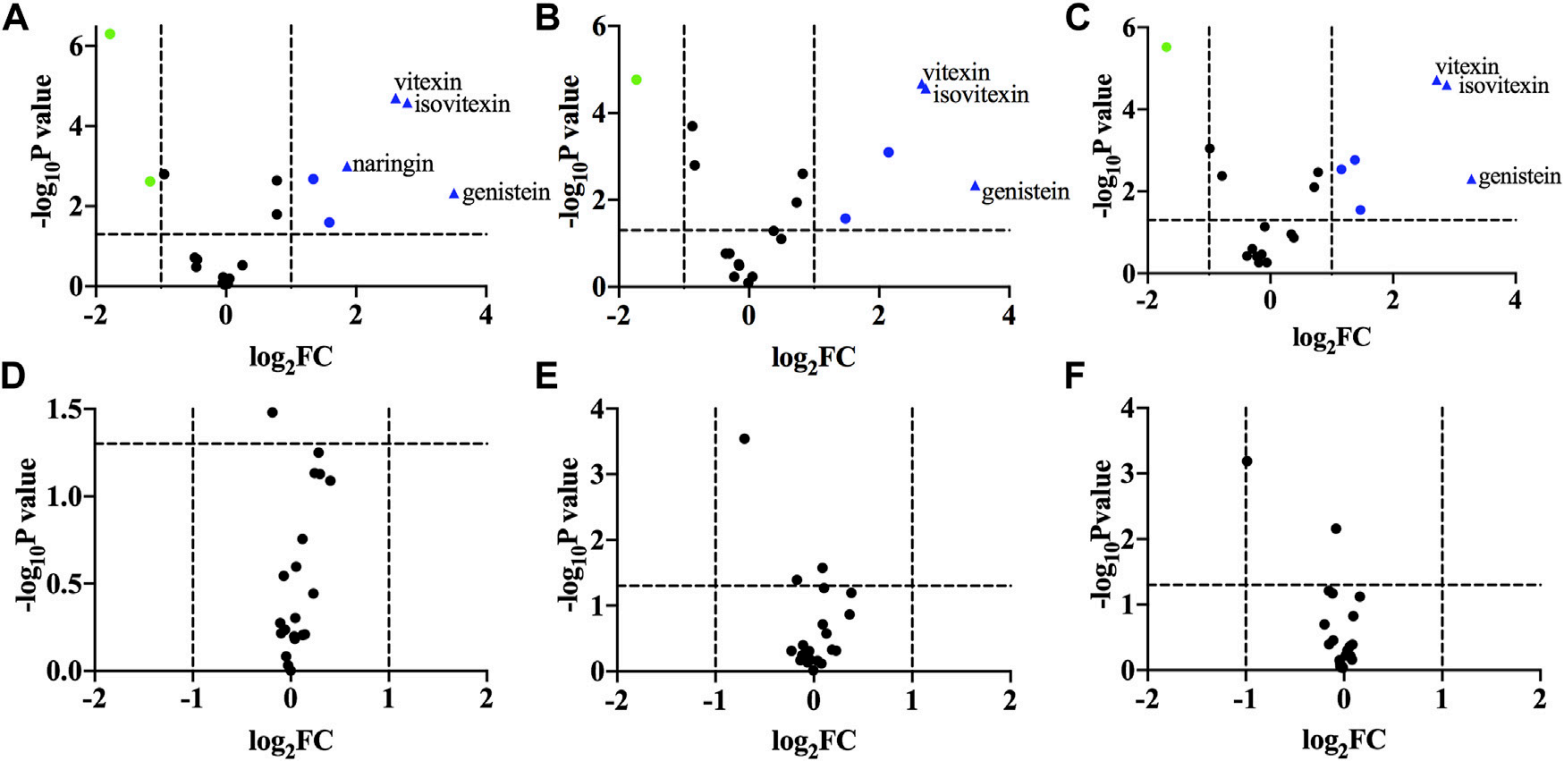
Volcano plot. **(A)** a vs. b; **(B)** a vs. c; **(C)** a vs. d; **(D)** b vs. c; **(E)** b vs. d; **(F)** c vs. **(D) (A)** represents bio-enzymatic method; **(B)** represents reflux method; **(C)** represents ultrasonic method; **(D)** represents ultrasound-assisted enzymatic method.

**FIGURE 4:**
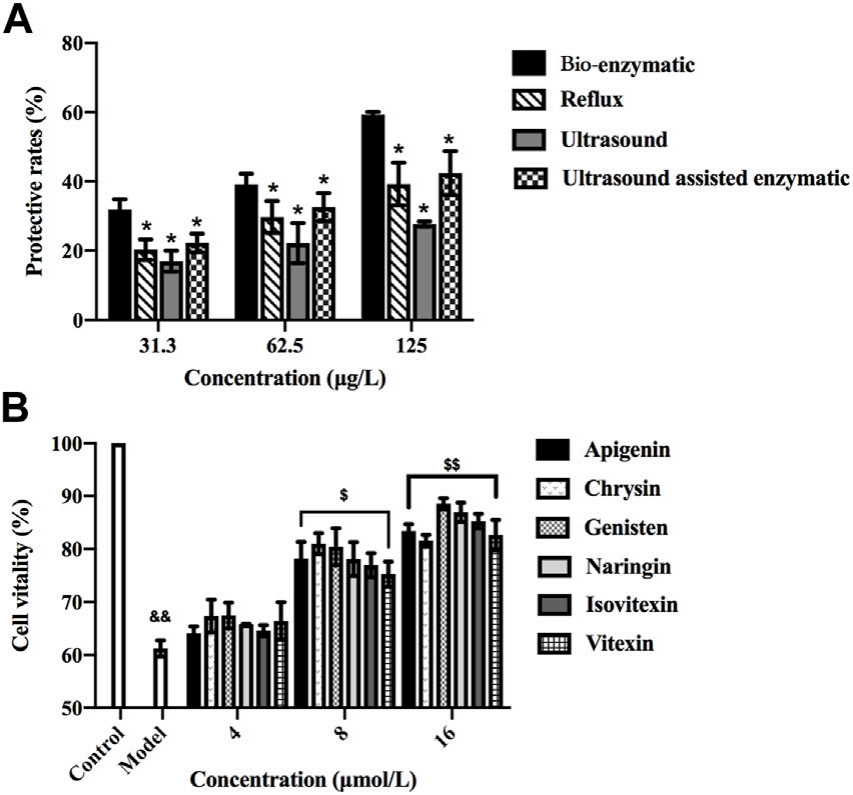
**(A)** Hepatoprotective effects of the total flavonoids extracted by reflux, ultrasonic, ultrasound-assisted enzymatic method (TFH) and bio-enzymatic method (Ey-TFH). *p < 0.05, compared with Ey-TFH. Data of Figure 4A were submitted simultaneously to Fourth International Scientific Conference” Alternative Energy Sources, Materials and Technologies (AESMT′21)"14th June 15th June 2021, Ruse, Bulgaria. **(B)** Cell viability after exposure to various concentrations of six differential components. && *p* < 0.01, compared with the control group; $ *p* < 0.05, $$ *p* < 0.01, compared with the model group.

A volcanic map was drawn to present the individual components that contributed to group classification in the next step. The results are depicted in Figure 3. The *x*-axis was the logarithm (base 2) of fold changes (FC). The *y* axis was the negative logarithm (base 10) of *p*-values (as it has been calculated in Table 4). Two vertical dashed lines represented FC < 2 or FC > 2, respectively. The horizontal dashed lines indicated *p* = 0.05. The upper layer of Figure 4 shows a comparison between TFH and Ey-TFH. Green or red represented significantly decreased or increased components in terms of FC and *p-*value, respectively. Red triangles represented components (genistein, isovitexin, naringin, and vitexin) that met the filtering criteria (FC > 3.5 and *p* < 0.01). The lower layer of Figure 3 compared extracts prepared by reflux, ultrasonic, or ultrasound-assisted enzymatic method. There was no significant difference in components among them.

After the comprehensive screening process, six representative differential components (vitexin, isovitexin, naringin, genistein, apigenin, and chrysin) were filtered for the subsequent cellular tests.

### 3.6 Hepatoprotective Effects of Ey-TFH, TFH and Six Differential Compounds *in vitro*

This part explored the hepatoprotective activities of TFH, Ey-TFH, and six further screened difference components. The hepatoprotective properties against H_2_O_2_ (200 μmol/L, 4 h) induced damage on HL-02 hepatocytes were studied. As shown in Figure 4A, the protective rates of TFH and Ey-TFH increased dose-dependently. Ey-TFH possessed the highest protection rate of all the tested concentrations compared with TFH (*p* < 0.05). According to Figure 4B, six differential components (vitexin, isovitexin, naringin, genistein, apigenin, and chrysin) all exerted significant protective effects on HL-02 hepatocytes at 8 and 16 μmol/L. In this light, it can be speculated that the hepatoprotective effect of six differential components might contribute to the superior protection of hepatocytes of Ey-TFH.

### 3.7 Ey-TFH Inhibited Hepatic Lipid Accumulation

**FIGURE 5:**
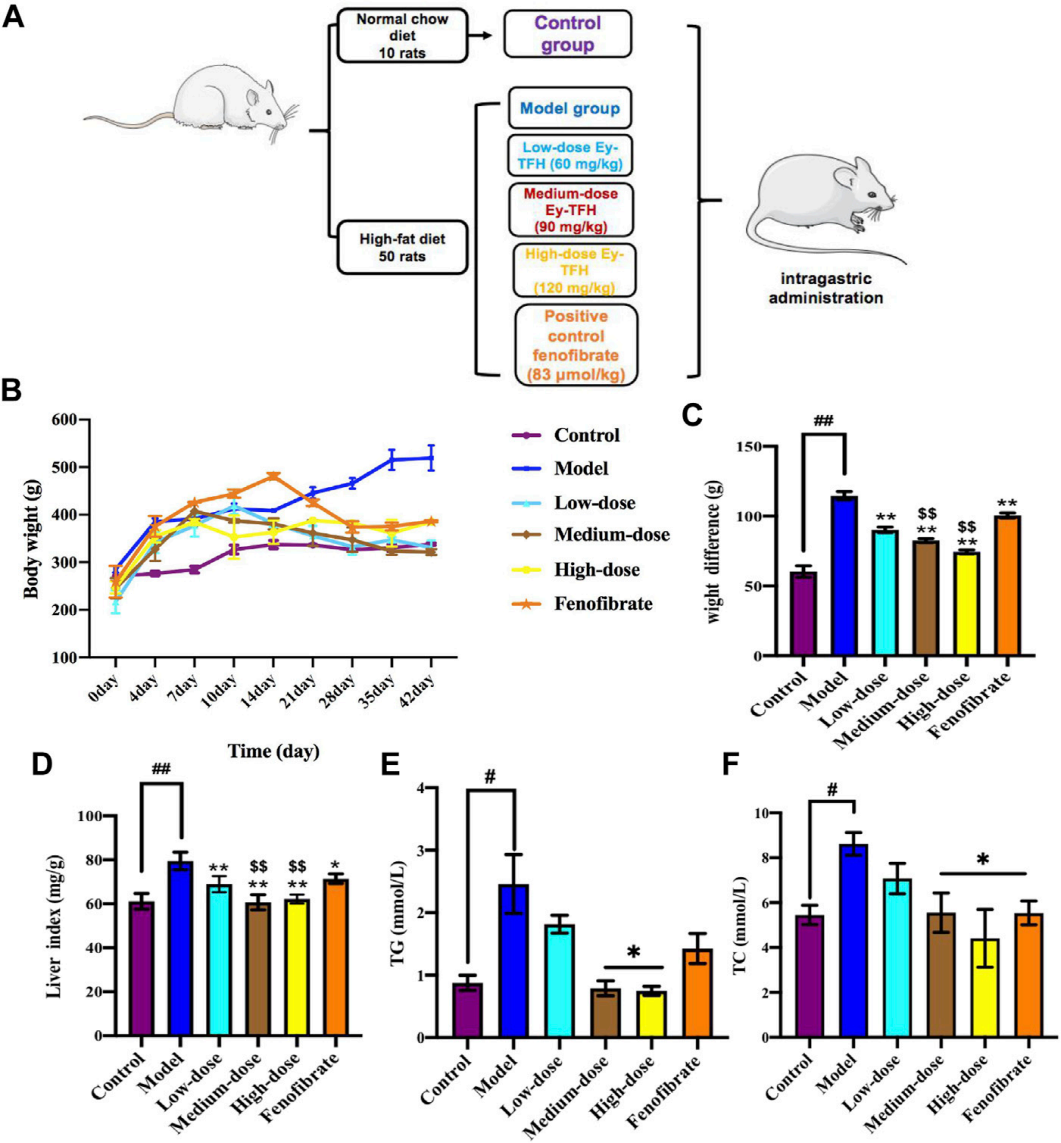
Effect of Ey-TFH on lipid accumulation. **(A)** The scheme of HFD - induced NAFLD model and Ey-TFH treatment. **(B)** Growth curve. **(C)** Weight difference. **(D)** Liver index. **(E)** TG contents. **(F)** TC contents. #*p* < 0.05, compared with the control group; *p < 0.05, compared with the model group; $$ *p* < 0.01, compared with the position control group.

Excess hepatic fat accumulates continuously at the early stage of NAFLD. This condition makes the liver extremely vulnerable to other damaging factors such as a plethora of oxidative stress, dysregulated hepatocyte apoptosis, and inflammation. These factors act together or separately, which would drive the progress from simple steatosis to nonalcoholic steatohepatitis. The therapeutic effect of Ey-TFH on NAFLD was investigated with a rat model of high-fat diet-induced obesity. We firstly investigated the weight gain and liver indexes. As shown in Figures 5C,D, Ey-TFH could reduce the risk of excessive weight gain and liver indexes. Next, we measured liver fat accumulation markers, serum TG and TC, after being treated with Ey-TFH. TC and TG contents ameliorated significantly after medium-dose and high-dose Ey-TFH treatment (Figures 5E,F). Taken together, Ey-TFH suppressed hepatic lipid accumulation.

### 3.8 Ey-TFH Protects the Liver in HFD—Induced NAFLD

**FIGURE 6:**
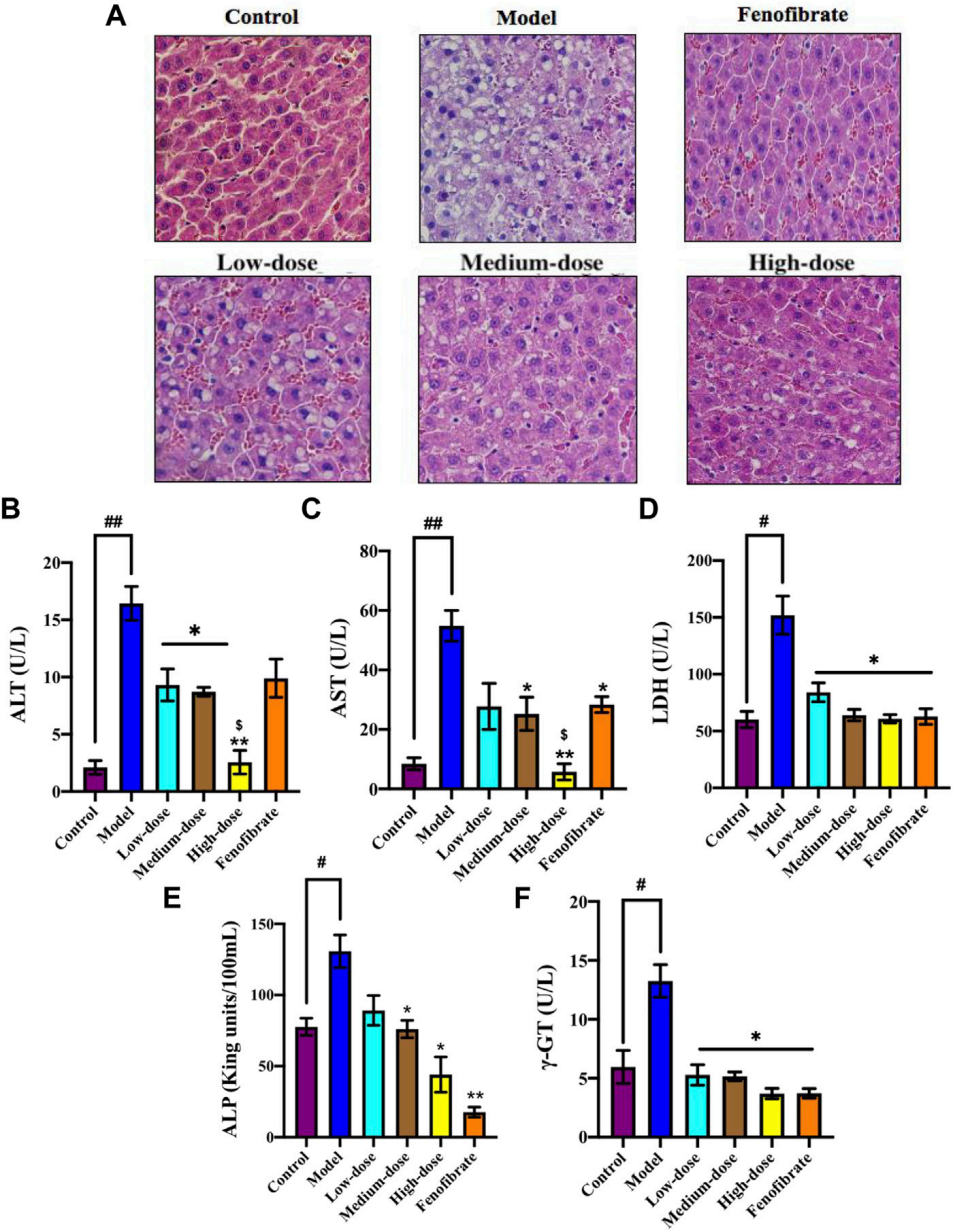
The protective effect of Ey-TFH on the liver. **(A)** HE staining. **(B)** ALT; **(C)** AST; **(D)** LDH; **(E)** ALP; **(F)** γ-GT. #*p* < 0.05, ##*p* < 0.01, compared with the control group; *p < 0.05, **p < 0.01, compared with the model group; $ *p* < 0.05, compared with the positive control group.

We tried to learn more about the improvement effect of Ey-TFH for NAFLD. Therefore, HE staining was used to assess the injury in liver sections. The result showed that Ey-TFH significantly reduced hepatic injury (Figure 6A). The liver injury markers such as ALT, AST, and LDH were measured. Large amounts of ALT, AST, and LDH were released from the damaged hepatocyte. The ALT, AST, and LDH sharply decreased after administration of Ey-TFH (Figures 6B–D). This showed that Ey-TFH significantly inhibited the elevation of liver injury markers. In addition, ALP and γ-GT, which were recognized markers of chronic inflammation, dramatically decreased in Ey-TFH groups (Figures 6E,F). These dates suggested that Ey-TFH protected the liver and blocked inflammation progress at the onset of NAFLD.

### 3.9 Ey-TFH Inhibit Lipid Peroxidation

**FIGURE 7:**
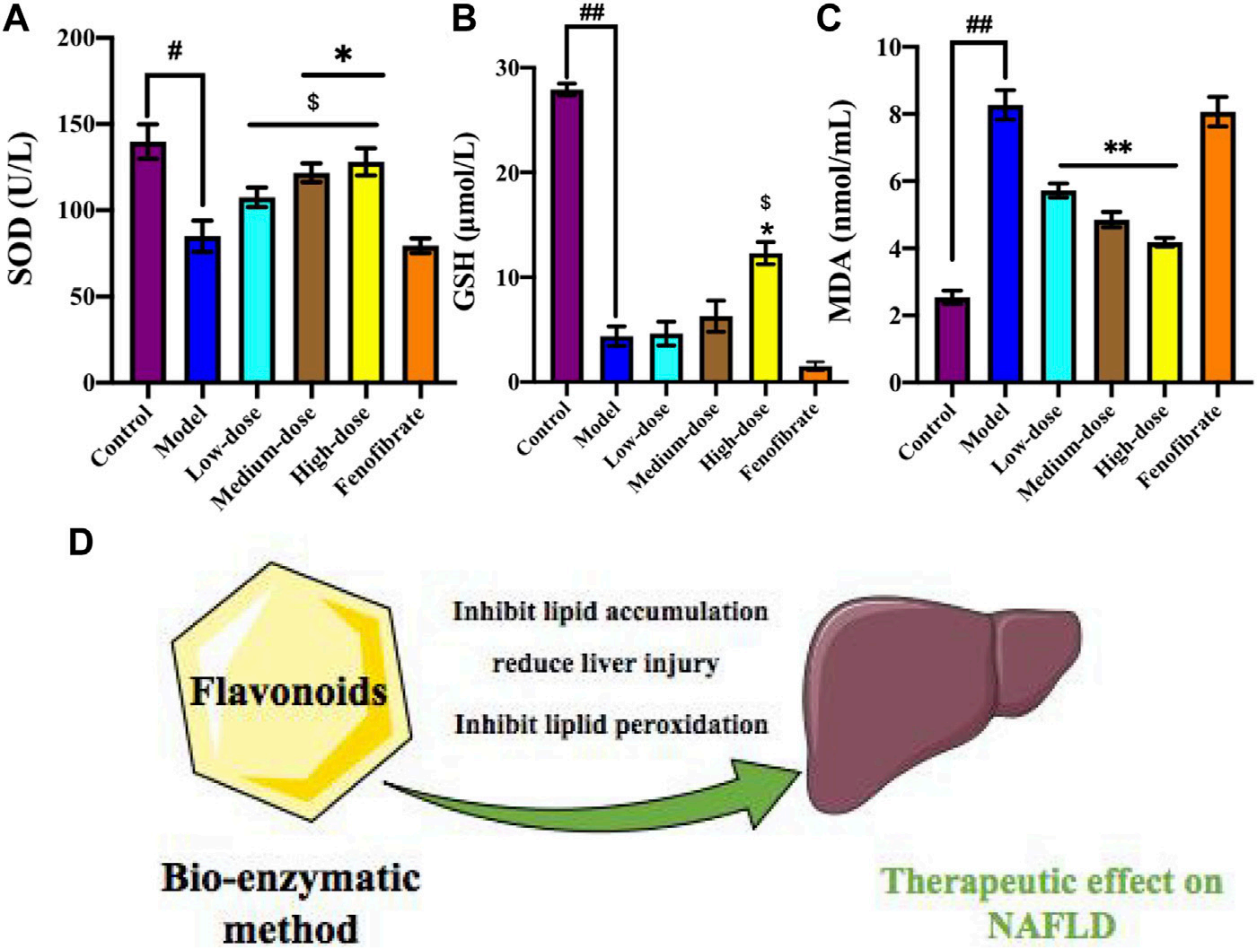
Ey-TFH inhibit lipid peroxidation. **(A)** SOD; **(B)** GSH; **(C)** MDA. **(D)** The therapeutic effect of Ey-TFH on NAFLD. #*p* < 0.05, ##*p* < 0.01, compared with the control group; *p < 0.05, **p < 0.01, compared with the model group; $ *p* < 0.05 compared with the positive control group.

After high-dose Ey-TFH administration, as shown in Figure 7, the activities of antioxidant enzymes, SOD, and GSH increased significantly. MDA contents, which represented the peroxidation levels of the membrane lipid, were reduced significantly by Ey-TFH intervention. The results clarified that Ey-TFH treatment could attenuate oxidative stress by reducing oxidative levels and promoting antioxidative processes.

## 4 Discussion

The total flavonoids, as important bio-active agents of *Scleromitron diffusum* (Willd.) R. J. Wang, possess high clinical medicinal value. The plant *Scleromitron diffusum* (Willd.) R. J.Wang belongs to the Rubiaceae family, so the total flavonoid yield of the plant was compared with other TCM plants from the Rubiaceae family, e.g., Uncaria rhynchophylla and Gardenia jasminoides Ellis. Our results in Table 2 found that *Scleromitron diffusum* (Willd.) R. J.Wang has a higher content of flavonoids than the other two plants, Uncaria rhynchophylla and Gardenia jasminoides Ellis (Lim and Lee, 2022 ; Shang et al., 2019). Moreover, flavonoids extracted by the bio-enzymatic method showed significant hepatoprotective activity both *in vitro* and *in vivo*. All these indicated that *Scleromitron diffusum* (Willd.) R. J.Wang may be a promising resource of flavonoids. It is also the reason why we chose *Scleromitron diffusum* (Willd.) R. J.Wang to study flavonoids. Thus it is necessary to explore the efficient extraction methods of flavonoids from *Scleromitron diffusum* (Willd.) R. J.Wang and more bio-actives should be detected. Various typical extraction processes such as water, microwave, and ultrasonic processed the total flavonoids. However, different extraction methods result in different contents and components of the total flavonoids. Research about the extraction methods mainly focuses on the extraction rate. Few studies focus on the differential flavonoid components obtained by different extraction methods. Research about the relationship between the differential components and bio-activity is also lacking. Therefore, a comprehensive method must be established to quantify the flavonoid content and screen out potential differential components. UPLC-MS/MS is a highly efficient and preferred technique for TCM (Nandan et al., 2021; Verma et al., 2021) and pharmacokinetics study (Chen et al., 2021; Zhang Z. et al., 2021; Lee et al., 2021; Wang et al., 2021; Zhang et al., 2022). In our study, the application combining UPLC-MS/MS and multivariate statistical methods was used to isolate and quantify flavonoids prepared by different methods, followed by differential components screened.

Compared with the traditional methods, using the bio-enzymatic method, cell walls were more gently and thoroughly destroyed by cellulase to promote the out-flow of intracellular flavonoids so that higher yields and more types of flavonoids could be harvested. That was consistent with our *in vitro* results. The cell results indicated that although Ey-TFH had no significant difference in total contents compared with TFH, Ey-TFH contained more active ingredients with good hepatoprotective effects, such as naringin (Li C. et al., 2020; Mu et al., 2020; Zhang R. et al., 2021; Zhao and Liu, 2021), vitexin (Inamdar et al., 2019; Duan et al., 2020; Yuan et al., 2020), and isovitexin (Hu et al., 2018). Thus, the flavonoids prepared by enzymatic extraction were more effective phytochemicals at protecting against H_2_O_2_-induced cellular injury. Furthermore, we found that Ey-TFH was efficient in rats. Ey-TFH treatment could inhibit HFD-induced weight gains and liver indexes, reduce lipid deposition, and improve the body’s antioxidant capacity. Collectively, we demonstrated that the total flavonoids extracted by the bio-enzymatic method could exert a beneficial effect on NAFLD. Improvement of antioxidant capacity may be the primary mechanism of NAFLD prevention by the total flavonoids. Hepatic steatosis is frequently accompanied by overwhelmed oxidative stress, a critical etiologic process of NAFLD. However, the underlying molecular mechanism of Ey-TFH hepatoprotective action is poorly understood, which will hopefully provide ideas for consecutive research.

In all, the combined application of UPLC-MS/MS and multivariate statistical methods guides the exploration of efficient and green extraction methods and offers valuable information on screening the bio-active compounds from TCM.

## 5 Conclusion

In the present work, a systematic method combining UPLC-MS/MS and multivariate statistical analyses was established to isolate and quantify the components of the total flavonoids from *Scleromitron diffusum* (Willd.) R. J. Wang, which were extracted by Luxembourg inventive bio-enzymatic method, reflux method, ultrasonic, and ultrasonic-assisted enzymatic method. Meanwhile, the differential components were also screened out. Compared with the other three methods, bio-enzymatic extraction could be more conducive to significantly increasing the contents of six flavonoids components (genistein, luteolin, luteolin-7-O-glucoside, naringin, isovitexin, vitexin) (*p < 0.05* or *p < 0.01*) and only three active components (apigenin, chrysin, kaempferol) were obtained. *In vitro* experiment showed that the six differential components (apigenin, chrysin, genistein, isovitexin, naringin, vitexin) all had hepatoprotection. The protective rate of Ey-TFH on HL-02 cells was significantly higher than those of TFH at the same concentration (*p < 0.05* or *p < 0.01*). At last, *in vivo* studies illustrated that Ey-TFH was an effective agent in NAFLD treatment. In conclusion, this study puts forward new ideas for extracting herb medicine and provides preliminary foundations for developing the total flavonoids prepared by Luxembourg inventive bio-enzymatic method as a promising agent for NAFLD treatment.

## Data Availability

The raw data supporting the conclusions of this article will be made available by the authors, without undue reservation.
