# Supplementary figures and images for: Isolation and Quantification of the Hepatoprotective Flavonoids From Scleromitron diffusum (Willd.) R. J. Wang With Bio-Enzymatic Method Against NAFLD by UPLC–MS/MS
Source: Front Pharmacol. 2022 Jun 13;13:890148. doi: 10.3389/fphar.2022.890148 (PMC9234865; doi:10.3389/fphar.2022.890148)

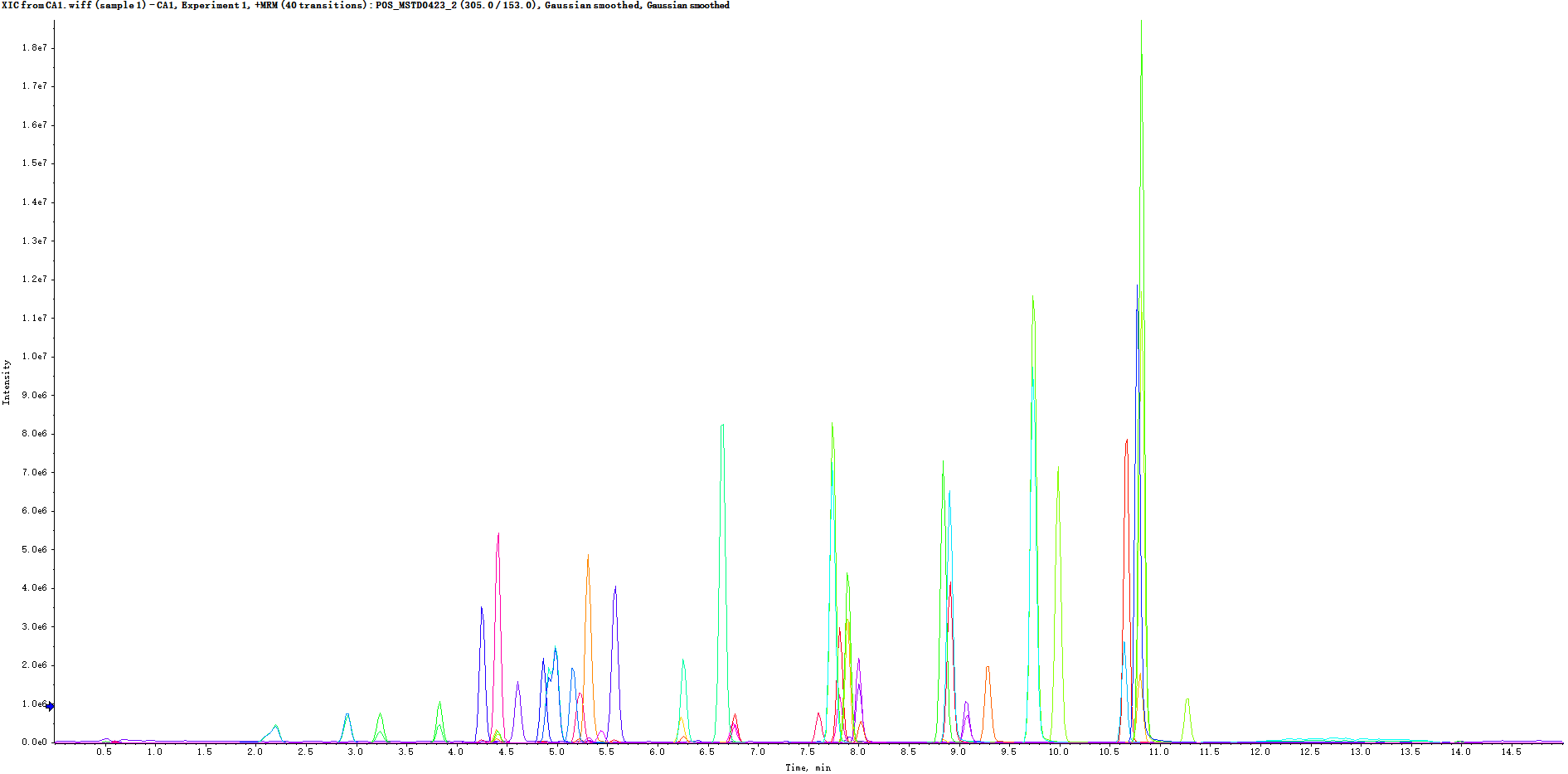

Supplement: Supplementary file 1 [file DataSheet2.zip › XIC.png]
